# UPLC-MS/MS-Based Metabolomic Profiling of Pollinated Loquat Fruits Reveals Cultivar-Specific Differences Between “Baihua” and “Dahongpao”

**DOI:** 10.3390/biology15141095

**Published:** 2026-07-08

**Authors:** Xin Sun, Zhuo Wang, Rui Shu, Yuli Qu, Yongsheng Li, Zhen Liu, Ping Zhao, Feng Chen, Ran Zhang, Cheng Cheng, Peng Hu, Yongjing Deng, Jian Liu, Junjun Dai

**Affiliations:** 1 Sericultural Research Institute, Anhui Academy of Agricultural Sciences, Heifei 230000, China; ahcsssx@163.com (X.S.);; 2 Xiaogan Public Inspection and Testing Center, Xiaogan 432100, China; 3 Huangshan City Animal Disease Prevention and Control Center, Huangshan 245000, China; 4 Huangshan City Animal Husbandry and Veterinary Technology Extension Center, Huangshan 245000, China

**Keywords:** loquat, *Apis cerana*, pollination, UPLC-MS/MS, phenolic acids

## Abstract

Bee pollination enhances the yield and fruit quality of loquats (*Eriobotrya japonica* Lindl.). However, the systemic effects of pollination on fruit metabolomics, particularly the metabolic differences among various cultivars following pollination, remain unclear. This study examined the metabolome of Baihua loquats following pollination by *Apis cerana* and compared it to that of unpollinated fruits. A total of 1964 metabolites were identified, including 334 differentially expressed metabolites. After analyzing ripe fruits of the white-fleshed loquat (Baihua, BH) and red-fleshed loquat (Dahongpao, DHP) following pollination, a total of 635 differentially expressed compounds were identified, with flavonoids, phenolic acids, and amino acids being the most abundant among the differentially expressed metabolites. This study further elucidated the biosynthetic pathways of phenylpropanoids, which account for the differences in the accumulation of BH and DHP in loquat following pollination. Consequently, these findings enhance our understanding of the overall metabolome of pollinated loquats, deepen our knowledge of polyphenolic metabolism in different loquat varieties, and provide new insights into the flavor differences between white-fleshed and red-fleshed loquats.

## Simple Summary

This study compared the metabolomic profiles of pollinated and unpollinated Baihua fruits following pollination by Apis cerana. Additionally, by analyzing differences in metabolites between ripe fruits of the Baihua and Dahongpao varieties after pollination, the study further elucidated the biosynthetic pathways of phenylpropanoid compounds and explained the differences in compound accumulation among different loquat varieties following pollination.

## 1. Introduction

The loquat (*Eriobotrya japonica* Lindl) belongs to the Rosaceae family. It is a subtropical fruit tree native to China that is rich in various phenolic acids and flavonoids. Known for its anti-inflammatory and antioxidant properties, it is widely cultivated around the world [1,2]. Loquats are partially self-pollinating plants, but cross-pollination can improve fruit set and quality [3,4]. Loquats bloom in the fall and winter and bear fruit in the spring, when the fruit ripens; their flowers face challenges posed by low winter temperatures and insufficient pollination [5]. Because loquats bloom in cold weather, they require pollinators adapted to low temperatures. Loquat flowers attract bees and other pollinators by secreting nectar rich in sucrose and releasing distinctive volatile aromatic compounds [6,7]. Due to their strong cold tolerance, *Apis cerana* have become one of the best pollinators for loquat flowers in winter [8]. The yield of loquat fruits significantly increased after pollination by bees, and their sugar content, moisture content, total soluble solids, total organic acids, and flavonoid content were all higher than those of unpollinated loquat fruits [9].

Ripe loquats of different varieties are generally classified as red-fleshed or white-fleshed based on the color of their flesh [10]. Loquat fruits are rich in various bioactive compounds, including phenolic acids, flavonoids, carotenoids, and vitamins; among these, phenolic acids are the primary metabolites in ripe loquats [11]. Differences in carotenoid accumulation result in white-fleshed and red-fleshed ripe fruits [12]. Ten bioactive compounds were identified in the two loquat varieties “Mogi” and “Tanaka,” and it was found that the phenolic acid chlorogenic acid is associated with fruit ripening [13]. Lignin, a phenolic compound, influences the lignification of the fruit flesh; particular attention is paid to the lignification of loquat fruit after harvest [2,14]. Current research on metabolites in loquat fruit has primarily focused on the functions of specific compounds, such as the antioxidant activity of phenolic acids and the influence of carotenoids on flesh color. The metabolic composition of loquat fruits following pollination by bees remains poorly understood. Therefore, to further investigate the metabolic composition of different loquat varieties after bee pollination and to understand the differences in metabolites between pollinated and unpollinated loquat fruits, dedicated research is still needed to report a comprehensive metabolite profile.

Broad-range targeted metabolomics based on ultra performance liquid chromatography–tandem mass spectrometry (UPLC-MS/MS) is a rapid and reliable analytical method widely used for the detection of plant metabolites [15]. Our study aims to investigate differences in metabolite formation in mature BH loquats under bee-pollinated and non-pollinated conditions, and to comprehensively characterize the metabolite profiles of BH and DHP loquat fruits from different varieties following pollination. The results comprehensively reveal the complete metabolite profiles of BH and DHP varieties and provide new evidence for metabolic changes in fruits following pollination by *Apis cerana*. The study provides a metabolic data foundation and new approaches for improving fruit quality through pollination biology.

## 2. Materials and Methods

### 2.1. Plant Materials

Two loquat cultivars Baihua (BH) and Dahongpao (DHP) were used in this study. Samples were collected from five-year-old loquat trees in Shexian County, Huangshan City, Anhui Province (29°84′23″ E; 118°06′45″ N). The loquat trees were grown in a clearing with a spacing of 3 m × 4 m. Standard horticultural methods, including pest and disease control, were used. For the unpollinated control group of loquat trees, physical isolation was achieved using 60-mesh insect netting throughout the entire loquat flowering period (Figure S1). The bee colony density was set at one colony of healthy *Apis cerana* per 0.1 hectare of forest land, ensuring that at least 10 bees were available for pollination on each loquat tree.

### 2.2. Sample Preparation and Metabolite Extraction

Six mature fruits were collected from five loquat trees, peeled and pooled into one biosample. At least three biosamples were collected from each loquat variety (≥18 fruits in total). All fruits were randomly collected on the same day in May 2024, during the loquat ripening period. Samples were stored at −80 °C before metabolite extraction. Using vacuum freeze-drying technology, place the biological samples in a lyophilizer (Scientz-100F, Scientz, Ningbo, China), then grind (30 Hz, 1.5 min) the samples to powder form by using a grinder (MM 400, Retsch, Haan, Germany). Next, weigh 50 mg of sample powder using an electronic balance (MS105DM, Mettler Toledo, Zurich, Switzerland) and add 1200 μL of −20 °C pre-cooled 70% methanolic aqueous internal standard extract (less than 50 mg added at the rate of 1200 μL extractant per 50 mg sample). Vortex once every 30 min for 30 s, for a total of 6 times. After centrifugation (rotation speed 12,000 rpm, 3 min), the supernatant was aspirated, and the sample was filtered through a microporous membrane (0.22 μm pore size) and stored in the injection vial for UPLC-MS/MS analysis.

### 2.3. Quality Control Measurements

Quality control (QC) samples were prepared by mixing equal volumes of loquat fruit samples of BH and DHP in order to assess the reproducibility of the mass spectrometry results. One QC sample was analyzed out of every 10 samples.

### 2.4. UPLC Conditions

The sample extracts were analyzed using an UPLC-ESI-MS/MS system (UPLC, ExionLC™ AD, https://sciex.com.cn/, accessed on 20 September 2024) and Tandem mass spectrometry system (https://sciex.com.cn/). The analytical conditions were as follows: UPLC column, Agilent SB-C18 (1.8 µm, 2.1 mm × 100 mm); the mobile phase consisted of solvent A, pure water with 0.1% formic acid, and solvent B, acetonitrile with 0.1% formic acid. Sample measurements were performed with a gradient program that employed the starting conditions of 95% A, 5% B. Within 9 min, a linear gradient to 5% A, 95% B was programmed, and a composition of 5% A, 95% B was kept for 1 min. Subsequently, a composition of 95% A, 5.0% B was adjusted within 1.1 min and kept for 2.9 min. The flow velocity was set as 0.35 mL per minute; The column oven was set to 40 °C; The injection volume was 2 μL. The effluent was alternatively connected to an ESI-triple quadrupole-linear ion trap (QTRAP)-MS.

### 2.5. ESI-Q TRAP-MS/MS

The ESI source operation parameters were as follows: source temperature 500 °C; ion spray voltage (IS) 5500 V (positive ion mode)/−4500 V (negative ion mode); ion source gas I (GSI), gas II (GSII), curtain gas (CUR) were set at 50, 60, and 25 psi, respectively; the collision-activated dissociation (CAD) was high. QQQ scans were acquired as MRM experiments with collision gas (nitrogen) set to medium. DP (declustering potential) and CE (collision energy) for individual MRM transitions were done with further DP and CE optimization. A specific set of MRM transitions were monitored for each period according to the metabolites eluted within this period.

### 2.6. Validation by Quantitative Real-Time PCR (qRT-PCR)

Candidate genes for qRT-PCR were identified based on the loquat transcriptome date from Wang et al., and then selected using DESeq2 with the following criteria: |Log2FC| ≥ 1.0 and FDR < 0.05 [16]. qRT-PCR primers for the selected genes were designed using Primer 5 (Premier Biosoft, Palo Alto, CA, USA). Detailed primer sequences are shown in Supplementary Table S4. qRT-PCR was performed on a CFX96 Real-Time System C1000 Thermal Cycler (Bio-Rad, Hercules, CA, USA) using the ArtiCanCEO SYBR qPCR Mix (Tsingke, Beijing, China) according to the manufacturer’s instructions. Relative gene expression was normalized to that of the reference gene, Actin. Gene expression levels were calculated using the 2^−ΔΔ CT^ method.

### 2.7. Statistical Analysis

All metabolites were identified using the MetWare database and quantified via multiple reaction monitoring (MRM). Metabolite data were analyzed using Analyst 1.6.3 software. Unsupervised PCA (principal component analysis) was performed by statistics function prcomp within R (www.r-project.org, accessed on 8 May 2025). The data was unit variance-scaled before unsupervised PCA. The HCA (hierarchical cluster analysis) results of samples and metabolites were presented as heatmaps with dendrograms, while Pearson correlation coefficients (PCCs) between samples were calculated by the cor function in R and presented only as heatmaps. Both HCA and PCC were carried out by R (base package 4.1.2) ComplexHeatmap. For HCA, normalized signal intensities of metabolites (unit variance scaling) are visualized as a color spectrum. For two-group analysis, differential metabolites were determined by VIP (VIP > 1) and absolute Log2FC (|Log2FC| ≥ 1.0). VIP values were extracted from OPLS-DA result, which also contain score plots and permutation plots, and was generated using R package MetaboAnalystR. The data was log transform (log22) and mean centering before OPLS-DA. In order to avoid overfitting, a permutation test (200 permutations) was performed.

## 3. Results

### 3.1. Phenotypic Differences in Loquats After Pollination

**Figure 1:**
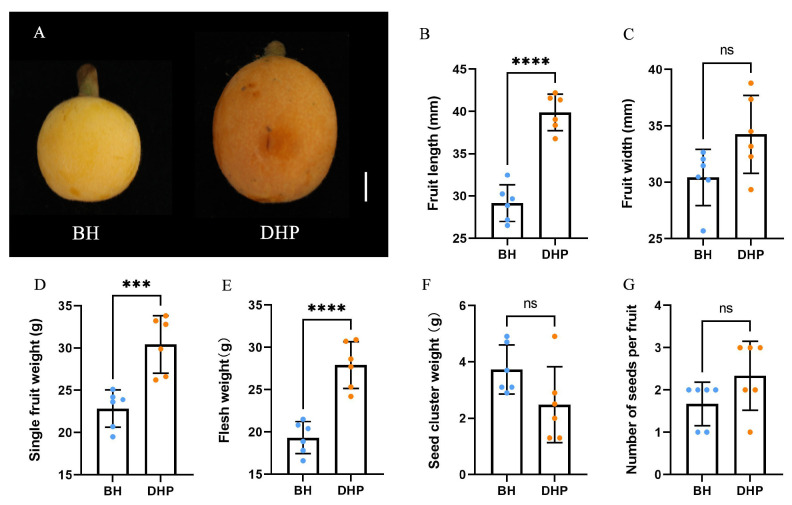
Phenotypic differences in the ripening of BH and DHP loquats. (**A**) Mature loquat fruits from BH (white-fleshed) and DHP (red-fleshed) cultivars. Bar, 1 cm. (**B**–**G**) BH and DHP loquat phenotypic data analysis. Statistical significance between different samples was assessed using a *t*-test. (ns indicates no significant difference, *** indicates *p* < 0.001, **** indicates *p* < 0.0001).

We conducted morphological analyses of BH loquat trees, some of which were pollinated by *Apis cerana* and others that were not. The results showed that, compared with fruits that had not been pollinated by bees, pollinated BH loquat fruits exhibited significant increases in individual fruit weight, flesh weight, and number of seeds. There were no significant differences in seed weight, fruit length, or fruit width; however, the fruits in the unpollinated control group were more irregular in shape (Figure S2). We then conducted morphological measurements on mature, post-pollinated loquat fruits of different varieties and found that mature BH loquat fruits were significantly smaller in size than DHP loquat fruits (Figure 1A). Specifically, the fruit length of BH loquats was significantly shorter than that of DHP loquats, while there was no significant difference in fruit width between the two (Figure 1B,C). Weighing of the fruits revealed that the average fruit weight of BH loquats was significantly lower than that of DHP (Figure 1D). We then separated the flesh and seeds of the ripe fruits for analysis and found that the difference in weight was primarily reflected in the flesh: the flesh weight of BH loquats was significantly lower than that of DHP loquats (Figure 1E), while there was no significant difference in seed weight between the two (Figure 1F). We counted the number of seeds separated and found no significant difference in seed count between the two varieties (Figure 1E).

### 3.2. Overview of Metabolites in Mature Loquats of Different Varieties Following Pollination

**Figure 2:**
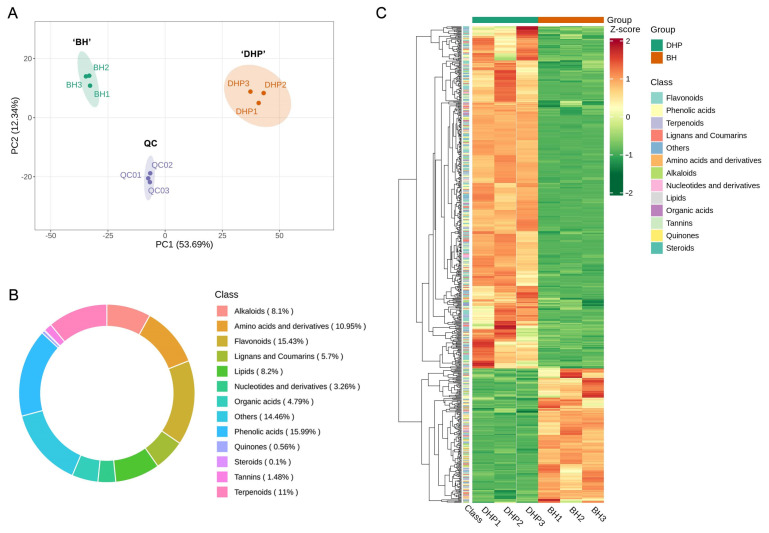
Overview of BH and DHP loquat metabolites. (**A**) PCA analysis of metabolites identified from BH and DHP. Equal volumes of BH and DHP fruit samples were mixed for use as a quality control (QC). (**B**) Pie chart showing the total number of metabolite categories. (**C**) Cluster analysis of metabolites from samples of BH and DHP. The color indicates the level of accumulation of each metabolite, from low (green) to high (red). The Z-score represents the deviation from the mean by standard deviation units.

Metabolites in BH were identified using UPLC-MS/MS to distinguish between pollinated and unpollinated samples. The analysis identified 1964 metabolites, including 334 differentially expressed metabolites. In mature BH loquats pollinated by the *Apis cerana*, 124 metabolites were upregulated and 210 were downregulated (Figure S3). Among the metabolites upregulated in BH loquats after pollination, the majority were phenolic acids, lignans, coumarins, amino acids, and terpenoids; in contrast, unpollinated loquat fruits exhibited relatively higher levels of flavonoid metabolites (Figure 2C). We then performed metabolomic analyses on BH and DHP loquats of different maturity stages following pollination, identifying a total of 1964 metabolites (Table S1). Among these, phenolic acids and flavonoids were the most abundant classes of metabolites detected, with 314 and 303 compounds identified, respectively (Figure 2B). We then performed an unsupervised principal component analysis (PCA) on the BH and DHP loquats and found that the principal components PC1 and PC2 accounted for 53.69% and 12.34% of the differences in metabolites between the two varieties, respectively (Figure 2A). The PCA results clearly separated the two loquat varieties, indicating significant differences in the metabolites present in the flesh of BH and DHP loquats. Heatmap analysis revealed significant differences in the composition of metabolites between BH and DHP loquats (Figure 2C).

### 3.3. Metabolites Differing Among Various Loquat Varieties Following Pollination

**Figure 3:**
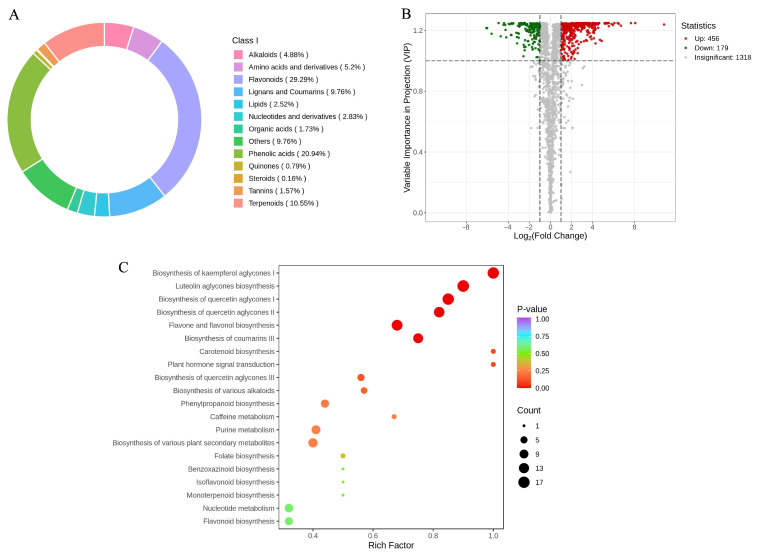
Metabolites specific to BH and DHP loquats. (**A**) Pie chart depicting the biochemical categories of the differential metabolites identified between BH and DHP. (**B**) Volcano plot of the 635 metabolites identified. Differential metabolites were defined as metabolites with fold change ≥2 or ≤0.5 in BH compared to DHP. A threshold of VIP ≥ 1.0 was used to separate differential metabolites from unchanged metabolites. (**C**) KEGG metabolic enrichment bubble plot of differentially expressed metabolites between BH and DHP.

Differentially expressed metabolites were identified using VIP (Variable Importance in Projection) > 1.0 and FC (Fold Change) ≥ 2 or ≤ 0.5, resulting in a total of 635 differentially expressed metabolites. Compared to DHP loquats, BH loquat pulp contained 456 more metabolites and 179 fewer metabolites. The differentially expressed metabolites included 186 flavonoids, 133 phenolic acids, 67 terpenoids, 62 lignans and coumarins, 62 other compounds, 33 amino acids and their derivatives, 31 alkaloids, 18 nucleotides and their derivatives, 16 lipids, 11 organic acids, 10 tannins, 5 quinones, and 1 steroid (Figure 3A,B). KEGG analysis revealed that the differences in the chemical composition of the ripe flesh of BH and DHP loquats were concentrated in metabolic pathways (34.9%) and the biosynthesis of secondary metabolites (28.19%), with a particularly high number of enriched pathways related to phenolic acids and flavonoids (Figure 3C and Figure S2).

### 3.4. Changes in Flavonoid, Phenolic Acid, Amino Acid, and Carbohydrate Content

We focus on the composition of compounds with a high proportion of certain compounds and the categories of metabolites of certain nutrients. Among the 334 differentially expressed metabolites identified in pollinated BH compared to unpollinated BH controls, 186 were flavonoids and phenolic acids. Among the 28 amino acid metabolites, the relative concentrations of 17 amino acids were significantly elevated in pollinated BH, including L-pyroglutamic acid, oxidized glutathione, and glycine-tryptophan. The relative content of monosaccharides and vitamin B1 in BH increased significantly after pollination (Table S2). A total of 635 differentially expressed metabolites were detected in BH and DHP varieties after pollination. Compared to the BH, the DHP exhibited 161 increases and 25 decreases in the relative content of flavonoids, and 98 increases and 35 decreases in phenolic acids. In contrast, the relative content of amino acids and their derivatives in DHP increased in only 10 cases and decreased in 23 cases compared to the BH. In the DHP loquat, the relative contents of five types of sugars—maltotriose, stachyose, sucralose, D-semicrystalline heptanose-7-phosphate, and ribose-5-phosphate—increased significantly, whereas in BH variety, only the relative contents of D-sucralose and D-glucosamine-1-phosphate increased significantly. The relative contents of four vitamins—*N*-glucosylnicotinic acid, pyridoxine, dehydroascorbic acid, and 2-*O*-glucosyl-L-ascorbic acid—were higher in DHP than in BH, whereas the relative content of procyanidins, a type of tannin compound, was significantly higher in BH than in DHP (Table S3).

### 3.5. Metabolic Pathways of Metabolites in Different Loquat Varieties After Pollination

**Figure 4:**
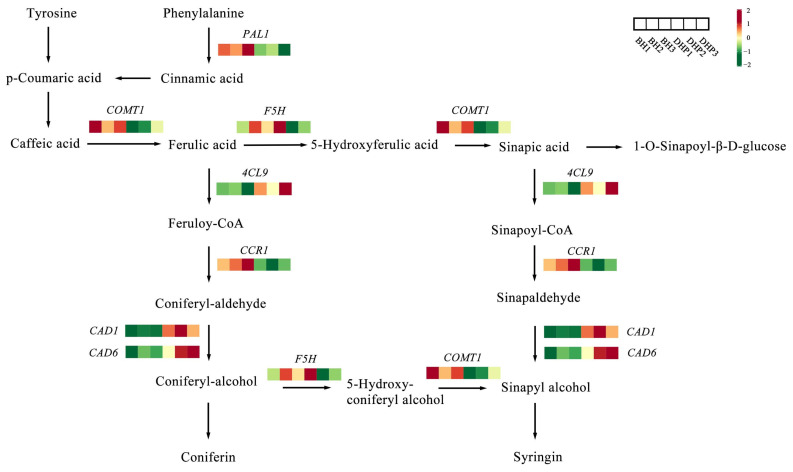
Metabolic pathways of phenolic acid metabolites in different loquat varieties after pollination.

To determine the effects of genes and metabolites on the metabolic pathways of phenolic acids and flavonoids among different varieties of BH and DHP, we analyzed the differentially expressed genes and metabolites associated with the phenolic acid metabolic pathway. The results showed that the *PAL1*, *COMT1*, and *CCR1* genes were significantly upregulated in BH loquats. The *4CL9*, *CAD1*, and *CAD6* genes were significantly downregulated in DHP loquats. The results indicate that in different varieties of loquat (BH and DHP), both metabolites and genes associated with the phenolic acid metabolic pathway have undergone changes (Figure 4). Among the phenolic acids, the levels of caffeic acid, sinapic acid, and 1-*O*-sinapoyl-*D*-glucose showed significant changes in the pathway. The content of DHP in the DHP loquat variety was significantly higher; we also observed corresponding changes in the levels of alcohol compounds derived from phenolic acids in the metabolic pathways. The levels of pinene, myristicin, and eugenol in the DHP loquat were significantly higher than those in the BH loquat (Figure S4).

## 4. Discussion

### 4.1. The Overall Regulation of Loquat Fruit Metabolism by Bee Pollination

Pollination plays a crucial role in maintaining ecological balance and improving the quality and quantity of agricultural production [17,18]. Honeybees are the most important pollinators among insects; for example, *Apis mellifera* L. provide 34% of the pollination services in the United Kingdom [19,20]. The role of bee pollination in enhancing fruit tree yield and fruit quality has been widely documented; however, there remains a lack of in-depth research on its systematic effects on fruit metabolomics, particularly the mechanisms underlying the differential responses among different varieties. Our results demonstrate that, compared to the unpollinated control group, a large number of differentially expressed metabolites were detected in the fruits of BH loquat pollinated by *Apis cerana*, and these metabolites were widely distributed across multiple metabolic pathways. Previous studies have found that loquats pollinated by bees exhibit significantly higher values for indicators such as individual fruit weight, total sugar content, and total soluble solids compared to loquats in the isolated control area [21,22]. After pollination, the levels of total flavonoids, total organic acids, and antioxidants in loquat flesh also changed significantly. We also found in BH loquats that not only were the phenotypic data of pollinated fruits superior to those of the isolation control group, but the relative content of phenolic acids and flavonoids—compounds with antioxidant activity—in the pollinated fruits had changed, and the fruits were richer in organic acids, tannins, terpenoids, vitamins, and other components. Research has found that similar trends are observed in apples and strawberries [23,24]. In terms of sugar metabolism, the sugar content of loquats increases significantly after pollination by bees [3,25]. Our results also demonstrate that the sugar content in BH loquats increases significantly following pollination by *Apis cerana*. As a result, the fruit shows a significant improvement in both flavor and quality after pollination.

### 4.2. Differences in Fruit Metabolism Between Two Loquat Varieties Following Bee Pollination

Previous studies have found differences in certain metabolites among different loquat varieties. Xu Hong et al. identified nine major phenolic acid compounds, including chlorogenic acid, ferulic acid, and caffeic acid, in six loquat varieties grown in China [26]. The major phenolic acids in ‘Chunhua No. 1’ loquats—chlorogenic acid, neochlorogenic acid, and pinitol—participate in the biosynthesis of phenylpropanoids, thereby influencing the formation of loquat flavor [27]. In terms of organic acids and sugars, BH loquats have lower levels of malic acid and carotenoids, but most of them have higher levels of glycerophospholipids [28]. Sugar and acid are beneficial nutrients that contribute to fruit quality, while carotenoids are responsible for the color of loquat peel and flesh [29,30]. We also found that the malic acid content in DHP loquats was significantly higher than that in BH loquats. Zhang Wenna et al. evaluated the phenolic compounds in seven loquat varieties grown in China and found that varieties rich in hydroxycinnamic acid (HCA) exhibited relatively high antioxidant activity, with the two red-fleshed varieties, Da hongpao and Luo yangqing demonstrating particularly high antioxidant potency indices [31]. Zou et al. identified 536 metabolites in Baiyu and Zaozhong No. 6, concluding that the flavor differences between varieties are primarily due to changes in the relative concentrations of carbohydrates, organic acids, amino acids, and phenolic compounds [32]. We also observed changes in the levels of organic acids, amino acids and their derivatives, and phenolic compounds in both the BH and DHP loquat varieties. Simultaneously, our results furnish supplementary insights into alterations in the relative quantities of compounds including flavonoids, terpenes, lignans, coumarins, alkaloids, and tannins.

### 4.3. Mechanisms Underlying Metabolic Differences in Fruits of Different Loquat Varieties Following Bee Pollination

Under bee-pollinated conditions, the enriched metabolic pathways in the fruit metabolome of the two loquat varieties, BH and DHP primarily involve phenolic acid biosynthesis, flavonoid biosynthesis, quercetin biosynthesis, and carotenoid metabolism. Previous studies have shown that carotenoid precursor genes and plastid-associated proteins (PAPs) play a major role in the carotenoid biosynthetic pathway [32,33]. We did not observe any significant changes in the carotenoid genes *DXS*, *VDE*, and *PSY1* in fully mature BH and DHP loquats, possibly because these carotenoid genes are active during the breaker stage (S4) of loquat growth and development. We found that *BCH* and *CYCB* were significantly upregulated in DHP loquats, whereas *CRTISO* was downregulated; this result is consistent with that reported by Zhang et al. [31,34]. After performing KEGG analysis on the metabolomics data of different loquat varieties (BH and DHP) following pollination, we found that the differentially expressed genes (DEGs) were primarily concentrated in the phenylpropanoid biosynthesis pathway within the phenolic acid metabolism pathway. In the anabolic metabolism of phenolic acids, the metabolism of major phenolic acid compounds, such as chlorogenic acid, ferulic acid, and caffeic acid, plays a central role [13]. Among these, *C4H1*, *4CL2*, *4CL9*, *HCT*, *CCoAOMT5*, *F5H*, *COMT1*, *CAD6*, and *POD42* in the “Snow White” (SW) loquat have been reported to be involved in the regulation of neochlorogenic acid synthesis and accumulation [35]. The expression levels of *PAL1*, *COMT1*, and *CCR1* in the BH loquat variety were significantly higher than those in the DHP variety, and *CCR* and *COMT* are key genes involved in lignin synthesis [36]. Lignin is formed through the polymerization of sinapyl, coniferyl, and p-coumaryl alcohols, a process in which the *CAD* gene plays a crucial role [37]. This study examined the expression levels of the *CAD1* and CAD6 genes in the CAD family and found that their expression levels were significantly higher in DHP loquats than in BH loquats, promoting the accumulation of coniferyl alcohol. This may be one of the reasons for the coarse flavor of DHP loquats. Zhang Kun and colleagues identified *4CL9*, *POD42*, *F5H*, and *β-glucosidase* as potential regulators of loquat phenolic compounds and fruit flesh texture [38]. Downregulation of *F5H* expression in Arabidopsis results in G-rich lignin, whereas high *F5H* expression leads to lower levels of the G-unit derived from coniferyl alcohol but higher levels of the S-unit derived from sinapyl alcohol [39]. We did not observe any differential expression of *F5H* in BH and DHP, but we did find differential expression of *COMT1* between the two strains. The *COMT1* expression level in BH loquats was significantly higher than that in DHP loquats, resulting in significantly lower relative contents of sinapic acid and sinapyl alcohol compared to DHP loquats. We speculate that *COMT1* may be negatively correlated with changes in the content of the lignin synthesis unit.

In this study, we built upon previous research on how *CAD* and *F5H* influence lignin synthesis and fruit texture in the phenylalanine biosynthetic pathway [38]. We elucidated the biosynthetic pathway of phenylpropanoids, which accounts for the differences in the accumulation of BH and DHP loquat phenolic acids following pollination, and clarified the roles of *PAL1*, *F5H*, *4CL9*, *CCR1*, *CAD1*, *CAD6*, and *COMT1* in this pathway. This study reveals the role of *COMT1* in the accumulation of sinapic acid and sinapyl alcohol in the phenylpropanoid biosynthetic pathway. Future work should focus on further investigating and validating the mechanisms by which these genes function in the biosynthesis of loquat phenylpropanol.

## 5. Conclusions

Using metabolomics, this study systematically compared, for the first time, the metabolic profiles of bee-pollinated and unpollinated (control) Baihua loquat fruits, as well as between the two loquat varieties following bee pollination, revealing the multidimensional regulatory effects of bee pollination on loquat fruit metabolism. At the same time, we conducted a further comparison of the metabolomes of different loquat varieties (BH and DHP) after pollination, revealing the distinct metabolite profiles between white-fleshed and red-fleshed loquats. This study elucidated the metabolic differences among various loquat varieties in the phenylpropanol biosynthetic pathway and identified *COMT1* as a potential target that influences the accumulation of phenolic metabolites, thereby affecting fruit flavor.

## Data Availability

All metabolomics data mentioned in this article have been uploaded to a public database. The link is available at https://www.ebi.ac.uk/metabolights/MTBLS13752 (accessed on 29 June 2026).

## Supplementary Materials

The following supporting information can be downloaded at https://www.mdpi.com/article/10.3390/biology15141095/s1, Figure S1: Control group of unpollinated white-flowered loquats. Use 60-mesh insect netting for physical isolation; Figure S2: Phenotypic differences between unpollinated and *Apis cerana* pollinated BH mature loquats; Figure S3: Overview of Metabolites in BH Loquats Before and After Pollination by *Apis cerana*; Figure S4: Comparison of the relative levels of metabolites in different loquat varieties after pollination; Table S1: Classes of non-volatile metabolites in Baihua and Dahongpao; Table S2: Significant differences in non-volatile metabolites between Baihua loquats pollinated by *Apis cerana* and unpollinated control Baihua loquats; Table S3: Significant differences in non-volatile metabolites between Baihua loquats and Dahongpao loquats; Table S4: Primers for real-time PCR.

## Abbreviations

The following abbreviations are used in this manuscript:

BH	Baihua
DHP	Dahongpao
UPLC-MS/MS	ultra performance liquid chromatography–tandem mass spectrometry
PCA	principal component analysis
VIP	Variable Importance in Projection
FC	Fold Change
HCA	hydroxycinnamic acid
PAPs	plastid-associated proteins
DEGs	differentially expressed genes
QC	quality control
