# Supplementary material for: UPLC-MS/MS-Based Metabolomic Profiling of Pollinated Loquat Fruits Reveals Cultivar-Specific Differences Between “Baihua” and “Dahongpao”
Source: Biology (Basel). 2026 Jul 8;15(14):1095. doi: 10.3390/biology15141095 (PMC13406099; doi:10.3390/biology15141095)
Supplement: Supplementary file 1 [file biology-15-01095-s001.zip › Supplementary Figures.pdf]

Supplementary Figures

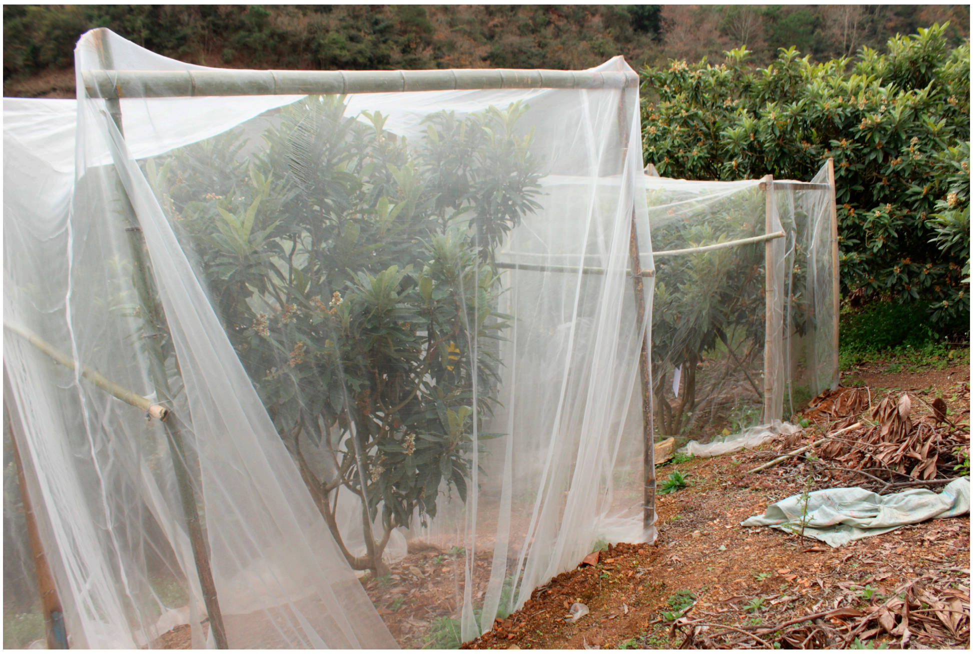

Figure S1. Control group of unpollinated white-flowered loquats. Use 60-mesh insect netting for physical isolation.

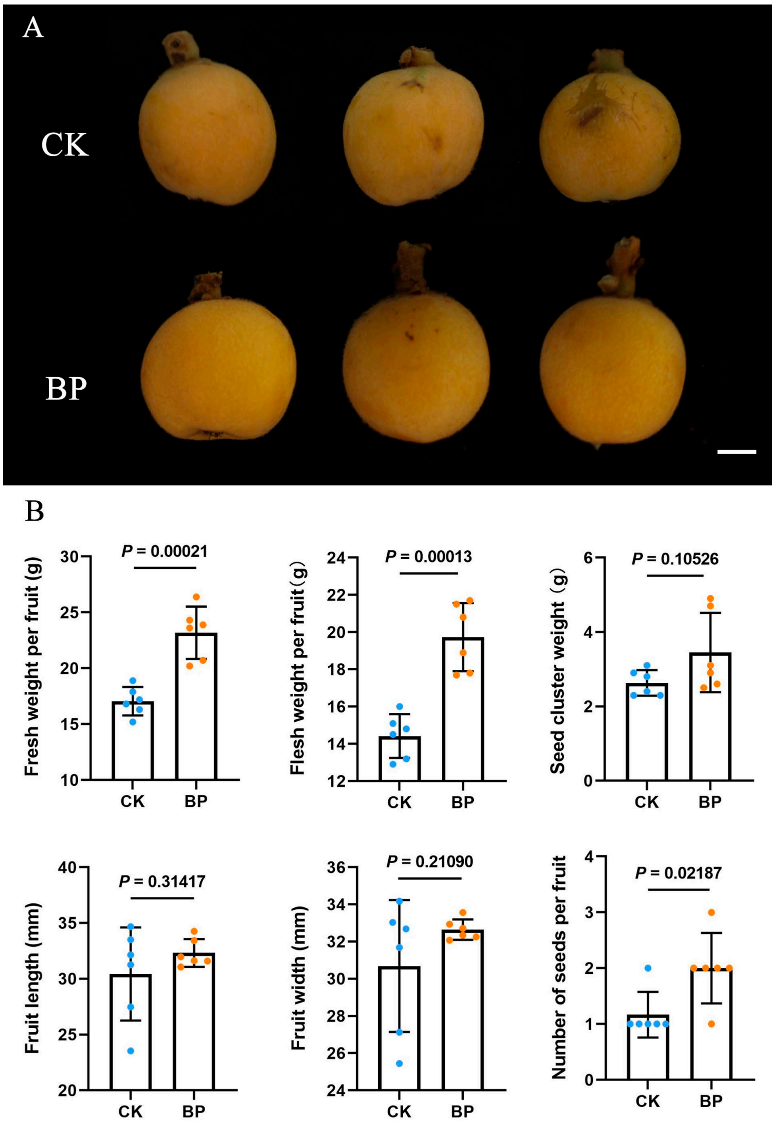

Figure S2. Phenotypic differences between unpollinated and *Apis cerana* pollinated BH mature loquats. (A) "CK" refers to the control group of mature loquats that have not been pollinated by *Apis cerana*, while "BP" refers to mature loquats that have been pollinated by *Apis cerana*. Bar, 1 cm. (B) BH and CK Loquat Phenotypic Data Analysis. Statistical significance between different samples was assessed using a t-test.

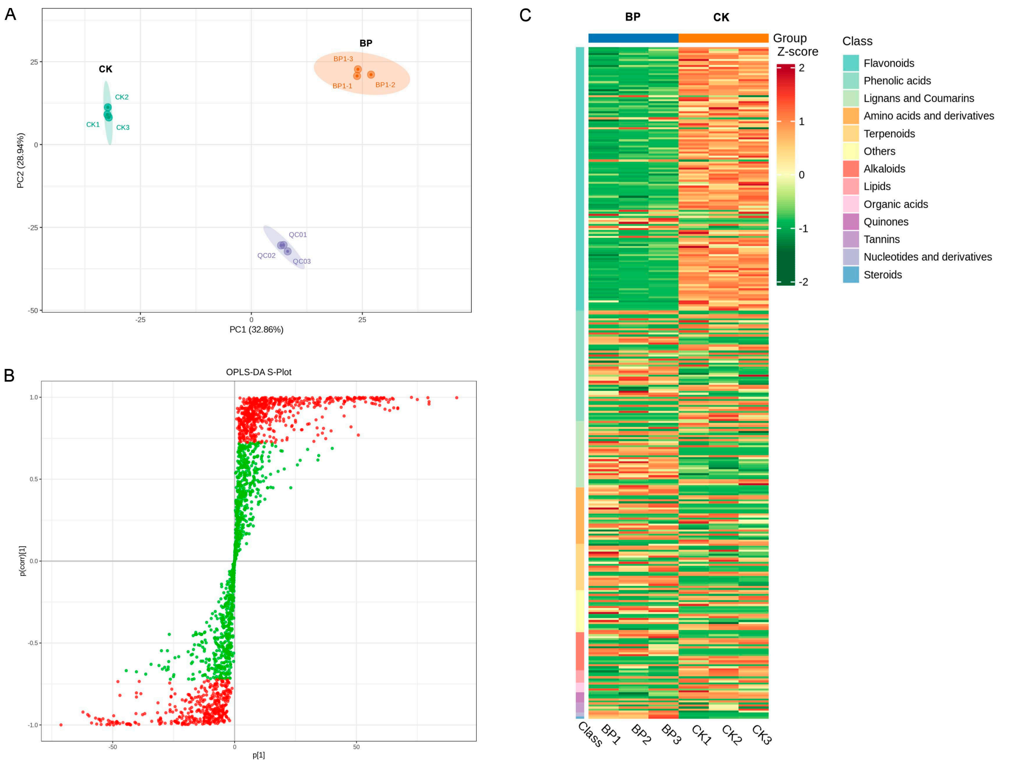

Figure S3. Overview of Metabolites in BH Loquats Before and After Pollination by *Apis cerana*. (A) PCA analysis of metabolites identified from unpollinated and *Apis cerana* pollinated BH mature loquats. Equal volumes of BH and CK fruit samples were mixed for use as a quality control (QC). (B) Volcano plot of the 334 metabolites identified. Differential metabolites were defined as metabolites with fold change ≥ 2 or ≤ 0. 5 in *Apis cerana* pollinated BH compared to CK. A threshold of VIP ≥ 1.0 was used to separate differential metabolites from unchanged metabolites. (C) Cluster analysis of metabolites from samples of unpollinated and *Apis cerana* pollinated BH mature loquats. The colour indicates the level of accumulation of each metabolite, from low (green) to high (red). The Z-score represents the deviation from the mean by standard deviation units.

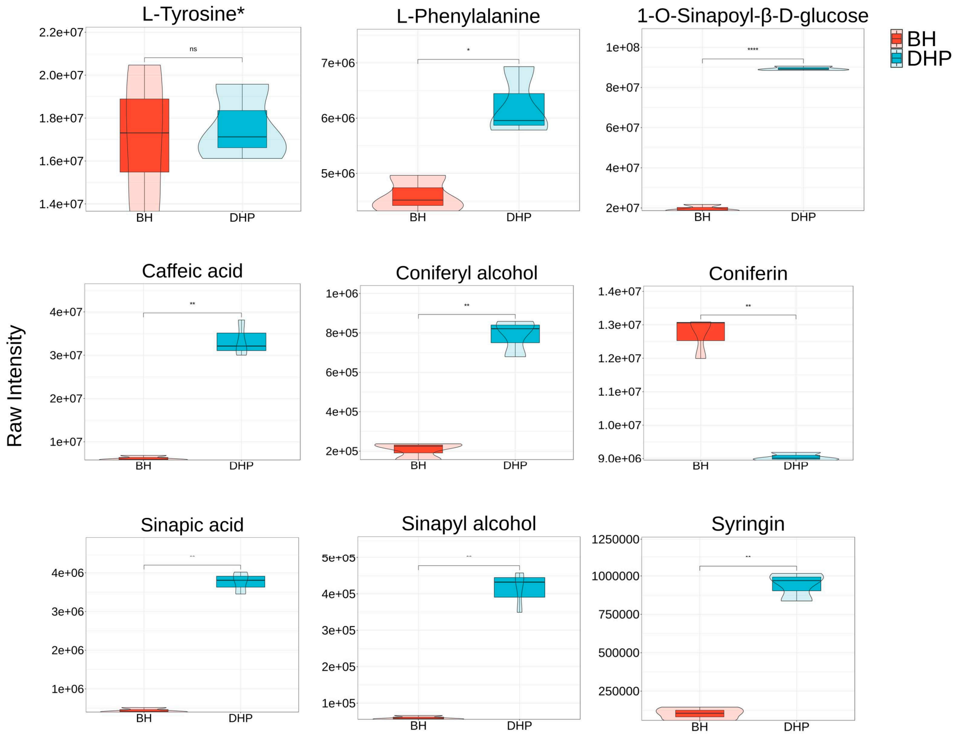

Figure S4. Comparison of the relative levels of metabolites in different loquat varieties after pollination. The expression level of *actin* was used to normalize the mRNA levels for each sample, with mRNA levels produced by Q-PCR expressed relative to *actin* level. Three replicates were performed for each sample.
